# Glycemic control and associated factors in patients with type 2 diabetes in Southwest Ethiopia: a prospective observational study

**DOI:** 10.1186/s12902-024-01795-y

**Published:** 2024-12-05

**Authors:** Aster Wakjira Garedo, Gorfineh Teshome Tesfaye, Rahel Tamrat, Evelien Wynendaele

**Affiliations:** 1 School of Pharmacy, Jimma University, Jimma, Ethiopia; 2 Jimma University Medical Center, Jimma, Ethiopia; 3 Jimma University School of Medical Laboratory, Jimma, Ethiopia; 4 Translational Research in Immunosenescence, Gerontology and Geriatrics (TRIGG) group, Ghent University Hospital, Ghent, Belgium; 5 Drug Quality and Registration (DruQuaR) group, Faculty of Pharmaceutical Sciences, Ghent University, Ghent, Belgium

**Keywords:** Glycemic control, Diabetes mellitus, Risk factors, Ethiopia

## Abstract

**Background:**

Diabetes, a known syndrome marked by hyperglycemia and glucose intolerance, is increasing at an alarming rate worldwide. Over half a billion people worldwide have DM, and most live in low- and middle-income countries. Poor glycemic control is a public health concern in type 2 diabetes mellitus. Glycemic control and identifying factors associated with poor glycemic control can help healthcare providers design programs that improve glycemic control and the quality of services provided to patients.

**Objectives:**

This study was designed to assess the level of glycemic control and associated factors in patients with type 2 diabetes in Jimma Medical Center, Southwest Ethiopia.

**Methods:**

This institution-based prospective observational study was conducted among 420 patients with type 2 diabetes at Jimma Medical Center’s diabetic clinics. A pretested structured interviewer-administered questionnaire was used to collect data, and a checklist was used to assess patient documents. The data were analyzed using SPSS version 26. The variables linked to poor glycemic control were investigated using binary logistic regression. Variables with *p* values less than 0.05 were considered statistically significant.

**Results:**

Six-month follow-ups were conducted among 420 patients with type 2 diabetes, among whom 220 (52.38%) were women. The median age of the participants was 54(IQR = 40–60 years old). The proportion of respondents with uncontrolled fasting blood glucose was 58.1%. Sex (AOR = 2.576, 95% CI [2.80-11.479], *P* = 0.001), age(≥ 60) (AOR = 2.024, 95% CI [1.794–4.646], *P* = 0.002), diabetes duration > 10 years (AOR = 3.036, 95% CI [2.616–8.306], *P* = 0.003), type 2 diabetes mellitus on insulin + oral antidiabetic (OADs) (AOR = 2.08, 95% CI [298-3.918], *P* = 0.004), obesity (AOR = 2.18, 95% CI [(1.218–4.218)], *P* = 0.003), diabetic complications (AOR = 3.193, 95% CI [2.324–6.05], *p* = 0.002) and poor self-care practices (AOR = 3.034, 95% CI [5.821–7.02], *P* = 0.005) were found to be significantly associated with poor glycemic control.

**Conclusion:**

At the Jimma Medical Center, the prevalence of poor glycemic control was high. Based on these findings, teaching and counseling provided by healthcare providers should focus on improving diabetes self-care activities, weight reduction, and diabetic complications to achieve good glycemic control.

**Clinical trial number:**

Not applicable.

## Introduction

Diabetes mellitus (DM) is a severe chronic illness that presents clinical challenges worldwide characterized by hyperglycemia and glucose intolerance [1], which arise from insufficient insulin production by the pancreas or inefficient insulin utilization by the body [2]. DM causes severe complications that impair various organ systems and substantially lower quality of life [3, 4]; over half a billion people worldwide are estimated to have DM [5]. The disease affects people of all ages, races, and socioeconomic backgrounds equally; however, its effects are most noticeable in low- and middle-income countries (LMICs), accounting for ~ 80% of all global diabetic cases [6]. It is estimated that 643 and 783 million persons between the ages of 20 and 79 will have DM by 2030 and 2045, respectively [5]. Type 2 diabetes mellitus (T2DM) is the most prevalent form of the disease, accounting for more than 90% of cases of diabetes [7–9].

Similar to the rest of sub-Saharan African countries, Ethiopia is experiencing a significant burden of DM, with increased prevalence, complications, mortality, and life-threatening disabilities. The World Health Organization (WHO) estimated the number of cases of diabetes in Ethiopia to be 800,000 in 2000 and projected that it would increase to 1.8 million by the 2030 year [3]. A previous study conducted in Ethiopia among patients with T2DM found that more than 80% of the patients had uncontrolled blood glucose levels [10]; only 5% of patients with DM had access to self-monitoring of blood glucose at home; none of them had HbA1c determination; and 75% of the patients required admission directly or indirectly due to uncontrolled DM, most importantly due to non-compliance with existing medications [12–16].

Although maintaining good glycemic control is the cornerstone of managing DM, which lowers healthcare costs, delays the onset of complications, and enhances the quality of life, only 50% of DM patients have their glucose under control globally, making diabetes care a continuing struggle [17–20], which can be achieved by measuring three different parameters: glycated hemoglobin (HbA1c), fasting plasma glucose (FPG), and postprandial glucose (PPG) [18, 21]. Currently, the burden of poor glycemic control and its complications increases significantly in Africa due to modernization, limited access to resources, healthcare, and education, and a Westernized lifestyle [18–23]. Despite the established facts that patients with DM benefit from hyperglycemia control, most fail to achieve adequate levels of glycemic control in LMICs, including Ethiopia [24–28].

Previous studies have investigated glycemic control and its associated factors in patients with type 2 diabetes in Ethiopia [11–16, 29, 30]. However, these studies were conducted using different study designs. Most utilized cross-sectional and retrospective studies, relatively small sample sizes, did not consider associated factors over longitudinal data, and some were conducted only in patients with type 2 diabetes on insulin regimens. Thus, a comprehensive investigation of glycemic control and associated factors utilizing a prospective follow-up study with a relatively larger sample size and the incorporation of important clinical and sociodemographic variables that can affect glycemic control will allow researchers to draw meaningful conclusions and identify key areas for intervention in diabetes management. Furthermore, this study helped to understand the extent of glycemic control and the impact of predictor variables on glycemic control in patients with T2DM in Ethiopia, one of the largest DM populations in sub-Saharan Africa. Therefore, this study aimed to determine glycemic control levels and associated factors in patients with T2DM treated at Jimma Medical Center in Southwest Ethiopia.

## Materials and methods

### Study setting, design, and period

We conducted an institution-based prospective observational study of patients with T2DM admitted to the diabetic clinic of Jimma Medical Center (JMC). This tertiary hospital, located in southwest Ethiopia, provides specialized care for patients with DM and serves a population of approximately 15 million. JMC offers various chronic follow-up clinics for both adult and pediatric patients. The endocrinology unit hosts two clinic visits per week specifically for patients with T2DM. The clinic operates twice a week, on Mondays and Tuesdays, to deliver integrated care to patients with diabetes. On average, 353 patients visited the diabetes clinic each month. The study was conducted from May to October 30, 2023.

### Population

The current study focused on all patients with T2DM who visited the chronic care clinic at JMC for follow-up. Participants included in the study were: Patients aged ≥ 15 years (considered adolescents at JMC), diagnosed with T2DM, underwent at least 6 months of follow-up, treated with either: oral antidiabetic (OADs) alone, Insulin alone, Combined therapy (OADs + Insulin), received care on monthly basis at JMC, and willing to participate in the study. Patients were excluded if they met any of the following conditions: diagnosed with psychological illnesses, pregnant women or those with gestational diabetes, recently diagnosed T2DM patients, or participants who expressed hesitation to participate.

### Sample size determination and sampling techniques

The sample size was determined using a single population proportion formula with a 95% confidence level. Considering a prevalence of poor glycemia among patients with T2DM (p) of 0.641 [21], and a desired sampling error (d) of 5%, the calculated sample size was 423. A systematic random sampling method was applied to select the study participants. The sampling interval (k) was calculated as approximately 5, derived from the total estimated number of patients with T2DM on treatment follow-up in six months (*n* = 2118) divided by the required sample size (*n* = 423). Consecutive sampling was used to include participants until the required sample size of 423 was reached.

### Data collection tools and procedures

A pretested structured questionnaire is used to ensure that the questions are clear and relevant to the study objectives. This questionnaire was adapted from validated instruments found in existing literatures [15–18]. The questionnaire was administered through face-to-face interviews, allowing for clarification of questions and immediate feedback from participants. A checklist is employed to assess patient documents, ensuring that all necessary information is collected systematically. This helps verify the accuracy of the data obtained from the interviews. A data abstraction format was used to gather information from the participants’ medical records. This format helps in collecting clinical data that may not be captured during interviews.

Based on the data gathering and longitudinal model in the study, variables are fixed or vary over time. Fixed variables such as Sociodemographic data: Age, gender, education level, occupation, and socioeconomic status; clinical data: medical and medication history, current medications and health status, and any existing comorbidities, behavioral data: lifestyle factors such as physical activity, smoking status, alcohol intake, khat chewing, and dietary habits. Anthropometric data: height and weight) are crucial for calculating the body mass index (BMI). Height was measured using a stadiometer, ensuring that participants stood upright with their buttocks, scapula, and head in contact with the measuring device. Weight is measured using a digital scale, with participants wearing light clothing and no shoes. BMI is calculated using the formula: BMI = Weight (kg)Height (m)2. The standard BMI classification is used to assess obesity, where: underweight: BMI < 18.5, Normal weight: BMI 18.5–24.9, Overweight: BMI 25–29.9, and obesity: BMI ≥ 30 [34]. Time-varying variables were insulin therapy, BMI, FBG, and the time of measuring blood glucose were collected monthly or daily by patients with T2DM who monitored their blood glucose level by themselves at home.

### DM self-care practice assessment

Diabetes Self-Care Activities (SDSCA) Scale: Developed by Toobert and Glasgow, this scale consists of 12 questions covering areas such as blood sugar monitoring, dietary habits, physical activity, foot care, and medication adherence [22]. In-person interviews were conducted with participants to gather responses to each question. The Diabetic Distress Score (DDS) developed by Fisher and collaborators [23], Average scores from the DDS were used to classify participants into two groups: moderate distress and no distress. Diabetic Discomfort Scale (DDS17): Participants rated their level of discomfort using this 17-item scale.

### Outcome measurement and validation

#### Primary outcome

The primary outcome of this study was glycemic control in patients with T2DM attending JMC diabetic clinic every 1–3 months follow-up appointments for 3 consecutive follow-ups. Glycemic control was confirmed by calculating the average fasting blood glucose (FBG) level.

#### Secondary outcomes

Secondary outcomes focused on complications associated with T2DM, defined by the presence of clinical signs and symptoms confirmed by a physician’s diagnosis recorded in the patient chart, along with relevant laboratory and imaging results indicating the development of new complications during follow-up.

#### Glycemic control monitoring methodology

This study assessed the rate of glycemic control using FBG levels, following the American Diabetes Association (ADA) recommendations. This approach is particularly relevant in resource-limited settings where HbA1c testing may not be routinely available [22].

#### Follow-up and categorization

Each patient was followed for a minimum of three months. FBG levels and other clinical data were recorded over three consecutive follow-up months and the average FBG level was used to categorize diabetes control into two groups: Controlled: 70–130 mg/dl, Uncontrolled: Below 70 mg/dl or > 130 mg/dl [22].

### Data management and quality assurance

To ensure the quality of data collected from patients with T2DM, several rigorous steps were implemented throughout the research process. Data collectors were trained thoroughly to familiarize themselves with the data collection instruments and procedures. To assess the correctness of the data collection tools, a pre-test was performed on 5% [18] of patients with T2DM. After data collection each day, the completeness and accuracy of the data were checked. This continuous monitoring allows for immediate identification and correction of issues, thereby enhancing the overall quality of data. The data collection instruments were initially written in English and were then translated into local languages, specifically Afan Oromo and Amharic. This step is essential to ensure that participants fully understand the questions being asked, which is particularly important in a diverse linguistic context. The back-translation of questionnaire by experts confirmed the accuracy of the tools. This process ensures that the meaning and context of the questions remain intact, thereby minimizing the risk of misinterpretation.

### Data analysis procedures

Epidata version 4.6, and SPSS version 26 was used for data entry and data analysis respectively. Descriptive statistics were used to describe the patients’ sociodemographic, clinical, and behavioral characteristics. To examine the relationships between categorical factors and blood glucose management, chi-square tests were performed. To investigate the causes of poor glycemic control, we conducted a multivariate logistic regression study. To identify independent factors affecting glycemic control, variables with *p* < 0.25 in the univariate logistic regression analysis were incorporated into the multivariate logistic regression model. At a 95% confidence level, variables with a *p*-value of less than 0.05 were deemed statistically significant.

### Operational definition and definition of terms

#### Fasting blood sugar

Blood glucose measured from venous blood after 8 h of overnight fasting or longer.

#### Adequate physical activity

The study participant followed the recommended exercise level for 3 or more days within the last seven days.

## Results

### Sociodemographic characteristics of the participants

Out of 423 responses, 420 (99.29%) were included in the data analysis. The exclusions consisted of one T2DM patient who declined to participate and two individuals with incomplete data. Of the 420 respondents, 220 (52.38%) were female. The average age of the study participants was 54 years (IQR: 40–60). Additionally, 283 (67.38%) of the respondents were married, and 119 (28.33%) had completed secondary school (Table 1).

**Table 1:** Socio-demographic characteristics of the study participants at Jimma Medical Center from May 1 to October 30, 2023, Jimma, Ethiopia

variables	Categories	Frequency	Percentage
Sex	Male	200	47.62
	Female	220	52.38
Age	< 40	101	24.0
	40–49	78	18.57
	50–59	113	30.47
	≥ 60	128	26.9
Educational status	Illiterate	82	19.52
	Primary school	119	28.33
	Secondary school	101	24.0
	Collage and	130	30.95
Marital status	Single	76	18.1
	Married	283	67.38
	Widowed	39	9.28
	Divorced/separated	22	5.23
Social drug use	Alcohol	101	24.1
	Chew Khat	201	47.85
	Smoking	118	28.09
Occupation	Unemployed	123	29.3
	Employed	122	29.1
	Merchant	123	29.3
	housewife	52	12.38
Income	≤ 1000 birr	216	51.42
	> 1000 birr	204	48.57
Health Insurance	Insured	244	58.1
	Uninsured	176	41.9
Distance to the health facility	Nearby	167	39.8
	Distant	253	60.2

### Self-care behaviors of participants

Of the 420 study participants, 263 (62.61%) did not engage in adequate physical activity. Additionally, 354 (84.28%) participants did not take accurate blood glucose measurements, whereas 241 (57.38%) were not adherent to a healthy eating pattern. However, 361 individuals (85.95%) reported taking their medication as directed by their healthcare providers (Table 2).

**Table 2:** Summary of diabetic self-care activities (SDSCAs) of the study participants at Jimma Medical Center, Ethiopia

Variables	Categories	Frequency	Percentage
Compliance with the general diet program within the last 7 days	> 3 days (adequate)	179	42.61
	0–3 days (inadequate)	241	57.38
Compliance with the foot care program within the last seven days	> 3 days (adequate)	316	75.24
	0–3 days (in adequate)	104	24.76
Physical exercise within the last 7 days	>3 days (adequate)	263	52.61
	0–3 days (inadequate)	157	37.38
Compliance with blood sugar testing within the last 7 days	> 3 (adequate)	66	15.71
	0–3 (inadequate)	354	84.28
Compliance with medication within the last 7 days	7 days (adequate)	361	85.95
	< 7 days (inadequate)	59	14.0

### Knowledge, behavioral, and clinical characteristics of the respondents

Regarding diabetes treatment, 115 (27.38%) of participants were not familiar with hyperglycemia and hypoglycemia symptoms, and 307(73.1%) were unaware of their target blood glucose levels. Among the respondents, 32 (71.9%) had fewer than three annual clinic follow-ups. The median duration of diabetes was 11 years (IQR: 5–17). Notably, all 420 respondents (100%) were receiving medication for their diabetes. Of participants using diabetes medication, 242 (57.61%) were reported using only oral antidiabetic (OADs) (Table 3).

**Table 3:** Knowledge, behavioral, and clinical characteristics of type 2 DM patients at Jimma Medical Center, Ethiopia

Variables	Categories	Frequency	Percentage
Ever attended diabetic education	Yes	220	52.38
	No	200	47.61
Number of follow-up visits to a diabetic clinic per year	≤ 3	302	71.9
	> 3	118	28.1
Number of diabetic education sessions ever attended (*n = 315*	1–2 times	174	55.23
	≥ 3 times	141	44.76
Knowledge of target blood glucose levels	Yes	113	26.9
	No	307	73.1
Knowledge of hyperglycemia signs and symptoms	Yes	305	72.61
	No	115	27.38
Alcohol consumption	Yes	110	26.19
	No	310	73.8
Smoking	Yes	108	25.71
	No	312	74.28
Duration of diabetes	< 5 years	94	22.38
	5–10 years	123	29.28
	> 10 years	203	48.33
Drug regimen	OADs	242	57.61
	Insulin	135	32.14
	Insulin and OADs	43	10.23
Body mass Index (kg/m2)	Normal (18.5–24.9)	79	18.8
	Overweight (25-29.9)	112	26.67
	Obese (> 30)	230	54.76
Blood pressure (SBP/DBP mm hg)	Optimal (< 130/80)	130	30.95
	Off-optimal (> 130/80)	290	69.1
FBS (mmol/L)	Normal range (4 -6.1)	176	41.9
	Hyperglycemia (> 6)	214	51.0
	Hypoglycemia (< 4)	30	7.1
DM complications	Neuropathy	56	13.3
	Nephropathy	113	26.9
	Retinopathy	41	9.8
	Hypertension	110	26.2
	Heart failure	12	2.9
	coronary heart disease	38	9,1
	cerebrovascular disease	33	7.9
	peripheral arterial disease	17	4.1
Number of complications	< 5	265	63.1
	≥ 5	155	36.9

### Magnitude of glycemic control

**Fig. 1:**
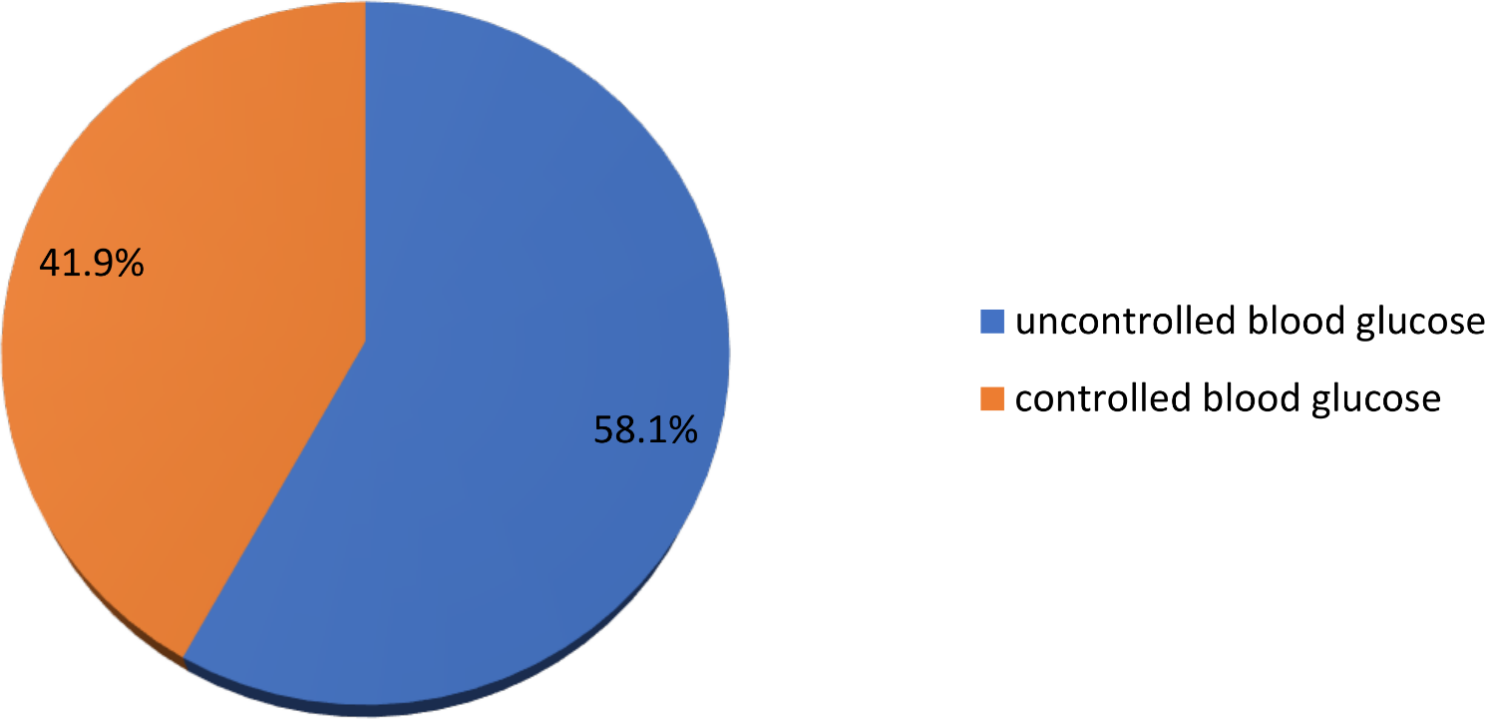
Magnitude of glycemic control among patients with type 2 diabetes at Jimma Medical Center, Ethiopia

Fasting blood glucose measurements taken over nine months during follow-up were used to assess glycemic control. The mean fasting blood glucose level recorded was 167.63 mg/dL ± 51.82 mg/dL, with minimum and maximum values of 43 and 312 mg/dL, respectively. Less than half (41.9%) of the patients achieved the American Diabetes Association’s recommended targets (Fig. 1).

### Trends in fasting blood glucose levels during follow-up

**Fig. 2:**
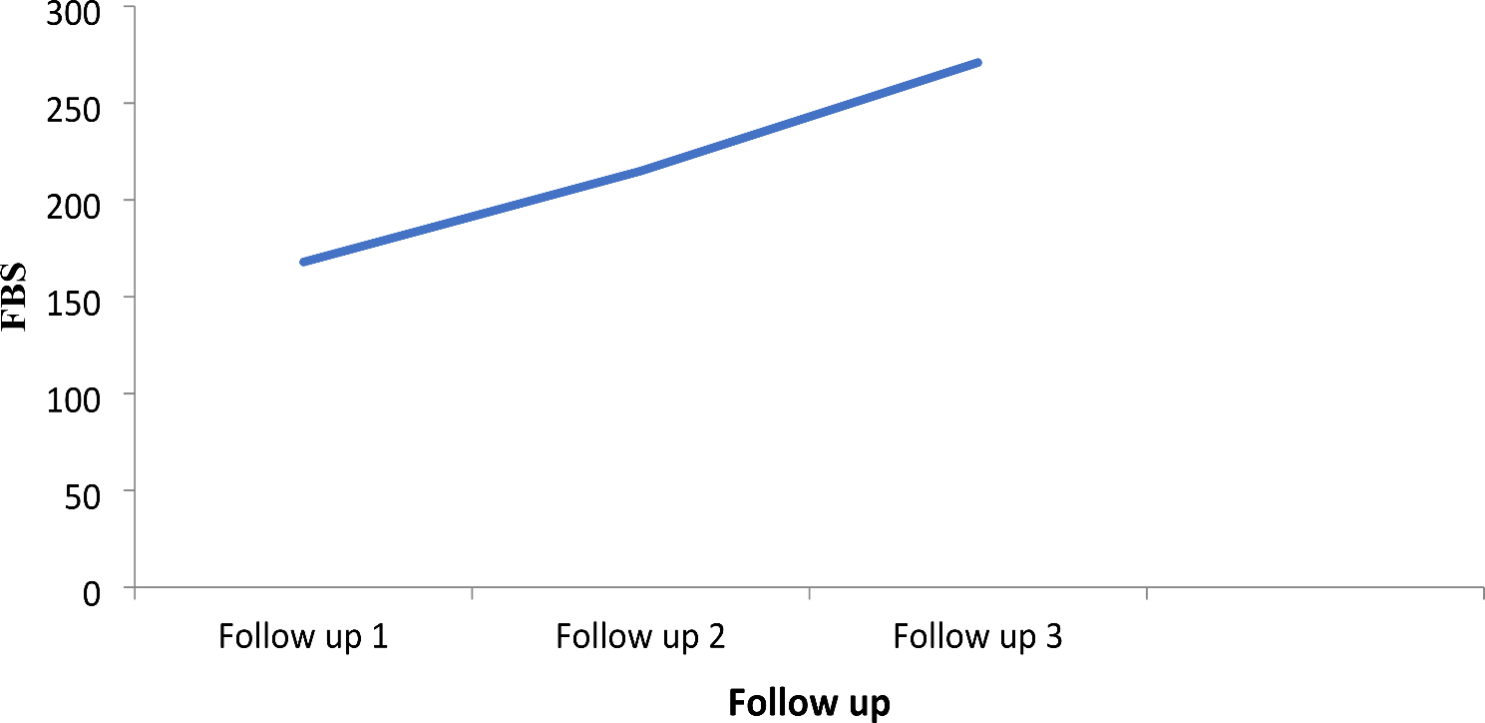
Trend of FBG in patients with type 2 diabetes in sequential measurements at Jimma Medical Center, Ethiopia

More importantly, the trend of fasting blood glucose (FBG) levels during follow-up showed an increasing level. From the beginning to the end of the study, the mean FBG level increased from 168 mg/dL to 271 mg/dL, indicating deterioration in patient conditions (Fig. 2).

### Factors associated with poor glycemic control

Bivariate logistic regression revealed that sex, age, duration of DM, drug regimens, BMI, alcohol consumption, and DM complications were associated with glycemic control. After controlling for potential confounding factors being female (AOR = 2.576, 95% CI [2.80-11.479], *P* = 0.001), older age (≥ 60) (AOR = 2.024, 95% CI [1.794–4.646], *P* = 0.002), alcohols (AOR = 2.48, 95% CI [2.391–8.342], *P* = 0.004), duration of DM > 10 years (AOR = 3.036, 95% CI [2.616–8.306], *P* = 0.003), T2DM on insulin + OADs drug regimen (AOR = 2.08, 95% CI [298-3.918], *P* = 0.004), obesity (AOR = 2.18, 95% CI [(1.218–4.218)], *P* = 0.003), DM complications (AOR = 3.193, 95% CI [2.324–6.05], *P* = 0.002), and poor self-care practices (AOR = 3.034, 95% CI [5.821–7.02], *P* = 0.005) were independent predictors of poor glycemic control (Table 4).

**Table 4:** *Factors associated with poor glycemic control in patients with T2DM at Jimma Medical Center*,* Ethiopia*

Variables	Categories	glycemic control		COR (95%CI)	*P* value	AOR (95% CI)	*P* value
		Good/ controlled	Poor/ Uncontrolled				
Sex	Male	70(39.7)	177(72.5)		1		1
	Female	106(60.3)	67(27.5)	3.697(1.088-939)	0.002	2.576(2.08–1.479)	0.001
Age	< 40	22(12.5)	110(62.5)		1		1
	40–49	36(20.5)	73(41.5)	1.56(0.786-3.023)	0.206	0.085(0.507-5.613)	0.29
	50–59	52(29.5)	91(51.7)	1.00(0.563-4.515)	0.117	1.014(0.071-12.186)	0.8
	≥ 60	65(36.9)	93(52.8)	1.400(0.946-0.383)	0.001	2.024(0.794-4.646)	0.002
Social drug use	Smoking	46(26.1)	56(22.9)		1		1
	Chew Khat	99(56.3)	102(41.8)	2.56(1.786-0.023)	0.34	1.924(2.704–5.626)	0.23
	Alcohol	31(17.6)	86(35.3)	1.25(0.563-4.515)	0.093	2.48(2.391–8.342)	0.004
Comorbidity	Yes	127(72.2)	100(41.0)	1.087(1.729-406)	0.86	2.229(0.794-5.646)	0.41
	No	49(27.8)	144(59.0)		1		1
Duration of diabetes	< 5 years	49(27.8)	45(18.4)		1		1
	5–10 years	62(35.2)	61(25.0)	1.50(0.857-6.373)	0.096	1.005(0.627-8.171)	0.38
	> 10 years	65(36.9)	138(56.5)	1.025(0.957-0.809)	0.002	3.036(2.616–8.306)	0.003
Drug regimen	OADs	69(39.2)	82(33.6)		1		1
	Insulin	64(36.4)	79(32.4)	1.492(0.762 − 2.92)	0.998	1.673(0.667-4.197)	0.189
	Insulin and OADs	55(31.4)	83(34.0)	3.843(0.805-0.223)	0.007	2.08(0.298-3.918)	0.004
Body mass Index (kg/m2)	18.5–24.9	50(28.4)	44(18.0)		1		1
	25-29.9	52(29.5)	75(30.7)	0.492(1.822-0.82)	0.22	1.623(2.925–5.323)	0.82
	> 30	74(42.1)	125(51.2)	2.843(1.805-223)	0.024	2.18(1.218–4.218)	0.003
Comorbidity	Yes	146(82.9)	194(79.5)	0.867(0.934-483)	0.45		
	No	30(17.1)	50(20.5)		1		
Number of Comorbidities	1	51(28.9)	42(17.2)		1		
	≥ 2	125(71.1)	202(82.8)	1.843(2.215-23)	0.37		
DM complications	Yes	138(30.9)	205(84.1)	1.023(1.523-563)	0.007	3.193(2.324–6.05)	0.002
	No	28(4.8)	39(15.9)		1		1
Poor Self-care practices	Yes	97(55.2)	102(41.8)	2.843(4.802-923)	0.002	3.034(5.821-0.025)	0.005
	No	79(44.8)	142(58.2)	1		1	

**COR**: crude odds ratio; **AOR**: adjusted odds ratio

## Discussion

This study assessed the magnitude of glycemic control and the factors affecting it among patients with T2DM at JMC in Southwest Ethiopia. The overall glycemic control of the participants was significantly below internationally recommended standards and guidelines. At JMC, fasting blood sugar levels were the sole metric used to monitor glycemic control, consistent with previous studies conducted in other regions of Ethiopia [19, 20]. This limitation stems from the unavailability of glycated hemoglobin (HbA1c) testing services and the high costs associated with HbA1c determination in government hospitals across Ethiopia. In contrast, developed countries primarily use the A1C test for glycemic management because it provides an average of glycemia over approximately three months [16, 27].

This study revealed that a high proportion of patients with T2DM had poor glycemic control. This finding is consistent with studies conducted in Tanzania [22] and Addis Ababa [19]. Additionally, our results indicate a significantly higher prevalence of poor glycemic control compared with the American Diabetes Association recommendations [24]. Conversely, our findings are lower than those reported in studies conducted in Tanzania [22], Saudi Arabia ([25], Ethiopia [14], Sudan ([27], India [26] and Northwest Ethiopia [23]. A possible explanation for this variation is that patients seeking advanced management were referred to JMC, the only tertiary hospital in Southwest Ethiopia, potentially affecting the overall glycemic control outcomes.

The FBG level during follow-up in patients with T2DM is a critical indicator of disease management and progression. In our study, mean FBG levels showed a concerning increasing pattern, rising from 168 mg/dL to 271 mg/dL. Similar trends have been observed in other studies [37–39]. This is because as T2DM progresses, patients often experience increased insulin resistance, pancreatic β-cells may fail to secrete adequate insulin, and poor self-care activities, including inadequate diet and lack of physical activity, can lead to poor glycemic control. The observed increase in fasting blood glucose levels among patients with T2DM highlights the need for proactive management strategies to prevent further deterioration of their condition. By focusing on education, monitoring, and lifestyle changes, healthcare providers can help patients achieve better glycemic control.

In our study, females were found to be 2.576 times more likely to have poor glycemic control than males. This finding is consistent with studies conducted in Kenya [31] and Koria [41]. These results showed potential gender differences in glycemic control, highlighting the need for a sex-specific approach to diabetes management. The less optimal glycemic control observed in women has not been adequately addressed in many diabetes management studies, making this finding critical for the effective management of women with T2DM. Possible explanations for this disparity include lower physical activity levels among females and specific feeding practices. Additionally, women with diabetes may be more prone to experiencing side effects from oral antidiabetic (OADs) as well as complications such as dyslipidemia and hypoglycemic events [42].

The study indicated that individuals aged 40 years and older with diabetes tend to have poorer glycemic control than their younger counterparts. These findings align with studies conducted in Tanzania [26] and Ethiopia [19]. One possible explanation for this observation is the less stringent glycemic control targets for older adults, which consider factors such as limited life expectancy, multiple comorbid conditions, and advanced microvascular or macrovascular complications. In such cases, the risks and burdens of intensive glycemic control may outweigh the benefits. However, this finding contradicts findings from a study in Ghana [30], in which older age was associated with better glycemic control. This variation may be attributed to the participants in Ghana having higher literacy levels, greater knowledge, and more experience in managing their diabetes, leading to better glycemic outcomes among older individuals.

In this study, the odds of poor glycemic control among patients with T2DM who consumed alcohol were 2.48 times higher than those of non-alcoholic individuals. Similar associations have been reported in studies conducted in Tanzania [22], Uganda [26], and Bosnia and Herzegovina [32], which also found a positive correlation between poor glycemic control and alcohol intake among patients with T2DM. Alcohol consumption is particularly detrimental to vulnerable populations, such as those with T2DM, given that it negatively impacts their ability to engage in self-care and affects vital body organs [33]. Research indicates that excessive alcohol consumption in patients with DM can lead to the accumulation of harmful substances, including acetic acid and acetaldehyde, in the bloodstream. This accumulation can result in severe complications, such as organ damage, dehydration, and increased blood pressure [16, 26].

In this study, overweight patients with T2DM were found to be 2.18 times more likely to have poor glycemic control than those with normal BMI. These findings agree with previous research conducted in South Africa [33], India [28], and Ethiopia [35]. This association may be explained by the fact that obese patients are more likely to experience poor glycemic control due to increased fat mass and visceral adiposity, which negatively impact insulin sensitivity and contribute to insulin resistance. In addition, some antidiabetic medications use may lead to weight gain in individuals with diabetes. Although metformin and thiazolidinediones are generally associated with weight neutrality or weight loss, other antidiabetic agents contribute to weight gain [36].

A significant proportion of patients with poor glycemic control receive a combination of OADs and insulin. The findings of this study are consistent with research conducted in Ghana [32], which showed that patients with poor glycemic control frequently require combination therapy to manage their diabetes effectively. Similar patterns were observed in Ethiopia [29, 40] and Malaysia [37]. The use of combination therapy involving OADs and insulin is a prevalent strategy among patients with T2DM who have poor glycemic control. This approach has been supported by evidence from multiple studies across different countries, emphasizing the need for personalized treatment plans that consider the unique challenges faced by individuals with T2DM. The current study showed that initial oral medications were often continued or added to the treatment regimen for patients exhibiting poor glycemic control. This tailored approach where healthcare providers adjust treatment plans according to individual patient needs and responses to therapy.

This study found that individuals with T2DM for more than 10 years exhibited a higher proportion of poor glycemic control than those diagnosed for less than 5 years. This observation is consistent with research conducted in Malaysia [37] and Iraq [38], which reported similar trends. This finding could be attributed to over time, the pancreatic β-cells responsible for insulin secretion may experience gradual failure. This reduction in insulin secretion significantly contributes to poor glycemic control in patients with long-term T2DM patients [39]. In addition, as T2DM progresses, patients often experience increased insulin resistance, which leads to reduced cell responses to insulin. This makes it increasingly difficult to manage blood sugar levels effectively. Furthermore, long-term diabetes management requires consistent monitoring of blood glucose levels and adjustments to treatment, exercise, and dietary habits. Patients with extended diabetes durations may find it increasingly challenging to adhere to these self-care activities, resulting in poorer glycemic control.

The findings of our study revealed a higher proportion of patients with poor glycemic control among those with complications than among those without. This finding aligns with research conducted in the United States [40], Ghana [36], and Malaysia [37]. Comorbidities and complications present significant challenges in diabetes management, including Pill Burden: Patients may face more medications, complicating treatment regimen adherence. The complexity of managing multiple health issues can lead to difficulties in maintaining consistent treatment, and additional health complications often result in higher healthcare costs. Moreover, these complications may be linked to underlying mechanisms such as β-cell damage and worsened insulin resistance. To achieve optimal glycemic control, diabetes-related complications and comorbidities must be effectively managed alongside the diabetes itself.

Moreover, this study found that poor self-care activities were 3.034 times more likely to be associated with poor glycemic control than good self-care activities. This finding aligns with previous research conducted in Kenya, Ethiopia, and Jordan [25, 41, 42], reinforcing the importance of effective self-care in managing T2DM. Effective self-care activities are crucial for achieving optimal glycemic control. Patients who engage in good self-care practices, such as regular monitoring of blood glucose levels, dietary management, and physical activity, are more likely to maintain better glycemic control. Therefore, providing comprehensive education on the importance of self-care activities, including effective monitoring of blood glucose levels and interpretation of results, is necessary. Implement support programs that encourage patients to adopt and maintain good self-care practices, which include group sessions, one-on-one counseling, and regular follow-up appointments to monitor patient progress and provide ongoing support and motivation for maintaining good self-care practices.

### Strengths and limitations of the study

This study employed a prospective observational study design and included a relatively larger sample size to ensure representativeness while investigating the association between glycemic control and various factors affecting it. Fasting blood glucose levels were used to assess glycemic control because of the unavailability of laboratory facilities for measuring glycated hemoglobin. The data were collected from a single health facility, limiting the generalizability of the results to a broader population. The observed high prevalence of poor glycemic control may exaggerate the true picture because the data were collected from patients attending a single hospital. Data obtained from self-report may be limited by self-reported data and recall bias can affect the validity of the findings and lead to inaccurate conclusions. To address the limitations associated with self-reported data and recall bias, we implemented mitigation strategies such as the use of external validation methods to compare self-reported data with objective measures by cross-checking self-reported medical, medication, or clinical data.

## Conclusion

This study found that poor glycemic control was significantly prevalent among patients with T2DM. To optimize glycemic control and enhance the quality of life for patients with T2DM, targeted interventions that focus on elderly patients, those with longer durations of diabetes and complications, obese patients, and those on insulin therapy should be implemented. The health sector should provide ongoing education that emphasizes behavioral lifestyle modification, including the importance of physical activity, self-blood glucose monitoring, and alcohol cessation. By focusing on these strategies, glycemic control in patients with T2DM can be enhanced, ultimately improving self-care activities and reducing the risk of diabetes-related complications.

## Data Availability

Readers who will require the data and materials of the current study can communicate and obtain the data from the corresponding author upon reasonable request.

## Abbreviations

AOR: Adjusted odds ratio
BMI: Body mass index
CI: Confidence interval
COR: Crude odds ratio
DM: Diabetes mellitus
FBS: Fasting blood sugar
HbA1c: Glycated hemoglobin
JMC: Jimma Medical Center
OADs: Oral antidiabetic
PPG: Postprandial glucose
SPSS: Statistical Package for the Social Sciences
SD: Standard deviation
T2DM: Type 2 diabetes mellitus
USD: United States Dollar

## References

[1] Chandio M, Kumar L, Khuwaja S, Memon UA, Bai K, Kashif M et al. Advances in the management of diabetes Mellitus : a focus on Personalized Medicine. 2023;15(8). 10.7759/cureus.43697 10.7759/cureus.43697 PMC10505357 37724233

[2] Aloke C, Egwu CO, Aja PM, Obasi NA, Chukwu J, Akumadu BO, et al. Curr Adv Manage Diabetes Mellitus. 2022;1–13. 10.3390/biomedicines10102436. 10.3390/biomedicines10102436 PMC9599361 36289697

[3] WHO. Global Report on Diabetes. Isbn. 2022;978(May):6–86. https://www.who.int/publications/i/item/9789241565257

[4] Yu J, Lee SH, Kim MK. Recent updates to clinical practice guidelines for diabetes Mellitus. Endocrinol Metab (Seoul). 2022;37(1):26–37. 10.3803/enm.2022.105. 35255599 10.3803/EnM.2022.105 PMC8901964

[5] IDF. IDF Diabetes Atlas IDF Diabetes Atlas. 2021.

[6] Banday MZ, Sameer AS, Nissar S. Pathophysiology of diabetes: an overview. Avicenna J Med. 2020;10(04):174–88. 10.4103/ajm.ajm_53_20. 33437689 10.4103/ajm.ajm_53_20 PMC7791288

[7] Care D, Suppl SS. Microvascular Complications and Foot Care : Standards of Medical Care in Diabetes d 2021. 2021;44(January):151–67. 10.2337/dc21-s011 10.2337/dc21-S011 33298422

[8] Mahgoub MO, Ali II, Adeghate JO, Tekes K, Kalász H, Adeghate EA. An update on the Molecular and Cellular basis of Pharmacotherapy in type 2 diabetes Mellitus. Int J Mol Sci. 2023;24(11). 10.3390/ijms24119328. 10.3390/ijms24119328 PMC10253663 37298274

[9] Talukder A, Hossain MZ. Prevalence of diabetes Mellitus and its Associated factors in Bangladesh: application of two-level logistic regression model. Sci Rep. 2020;10(1):1–7. 10.1038/s41598-020-66084-9. 32581295 10.1038/s41598-020-66084-9 PMC7314753

[10] Ahmad F, Joshi SH. Self-Care practices and their role in the control of diabetes: a narrative review. Cureus. 2023;15(7):e41409. 10.7759/cureus.41409. 37546053 10.7759/cureus.41409 PMC10402910

[11] Abera RG, Demesse ES, Boko WD. Evaluation of glycemic control and related factors among outpatients with type 2 diabetes at Tikur Anbessa Specialized Hospital, Addis Ababa, Ethiopia: a cross-sectional study. BMC Endocr Disord. 2022;22:54. 10.1186/s12902-022-00974-z. 35249547 10.1186/s12902-022-00974-z PMC8898656

[12] Fekadu G, Bula K, Bayisa G, Turi E, Tolossa T, Kasaye HK. Challenges and factors Associated with Poor Glycemic Control among type 2 diabetes Mellitus patients at Nekemte Referral Hospital, Western Ethiopia. J Multidiscip Healthc. 2019;12:963–74. 10.2147/jmdh.s232691. 31819470 10.2147/JMDH.S232691 PMC6878927

[13] Shita NG, Iyasu AS. Glycemic control and its associated factors in type 2 diabetes patients at Felege Hiwot and Debre Markos Referral Hospitals. Sci Rep. 2022;12(1):9459. 10.1038/s41598-022-13673-5. 35676526 10.1038/s41598-022-13673-5 PMC9177638

[14] Bitew ZW, Alemu A, Jember DA, et al. Prevalence of Glycemic Control and factors Associated with Poor Glycemic Control: a systematic review and Meta-analysis. INQUIRY: J Health Care Organ Provis Financing. 2023;60. 10.1177/00469580231155716. 10.1177/00469580231155716 PMC10071101 36852627

[14] Yahaya JJ, Doya IF, Morgan ED, Ngaiza AI, Bintabara D. Poor glycemic control and associated factors among patients with type 2 diabetes mellitus : a cross sectional study. Sci Rep. 2023;01234567891–10. 10.1038/s41598-023-36675-3. 10.1038/s41598-023-36675-3 PMC10267215 37316565

[16] Sendekie AK, Belachew EA, Dagnew EM. Rate of glycemic control and associated factors in patients with type 2 diabetes mellitus treated with insulin-based therapy at selected hospitals in Northwest Ethiopia: a multicentre cross-sectional study. BMJ Open. 2022;12:e065250. 10.1136/bmjopen-2022-065250. 36691186 10.1136/bmjopen-2022-065250 PMC9454077

[17] Lubaki F, Pierre J, Lubaki F, Omole OB, Francis JM. Glycemic control among type 2 diabetes patients in sub. Diabetol Metabolic Syndrome BioMed Cent. 2022;1–78. 10.1186/s13098-022-00902-0. Saharan Africa from 2012 to 2022 : a systematic review and meta analysis. 10.1186/s13098-022-00902-0 PMC9487067 36127712

[18] Davies MJ, Aroda VR, Collins BS, Gabbay RA, Green J, Maruthur NM, et al. Management of hyperglycemia in type 2 diabetes, 2022. A Consensus Report by the American Diabetes Association (ADA) and the European Association for the Study of Diabetes (EASD). Diabetes Care. 2022;45(11):2753–86. 10.2337/dci22-0034. 36148880 10.2337/dci22-0034 PMC10008140

[19] Lubaki JF, Omole OB, Msa J. Poor glycemic control : prevalence, factors and implications for the care of patients with type 2 diabetes in Kinshasa, Democratic Republic of the Congo : a cross-sectional study. 2023;(November):1–13. 10.3389/fcdhc.2023.1241882 10.3389/fcdhc.2023.1241882 PMC10699440 38076524

[20] Usman MS, Khan MS, Butler J. The Interplay between Diabetes, Cardiovascular Disease, and kidney disease. ADA Clin Compend. 2021;2021(1):13–8. 10.2337/db20211-13. https://www.ncbi.nlm.nih.gov/books/NBK571718/. 34279879

[21] Dimore AL, Edosa ZK, Mitiku AA. Glycemic control and diabetes complications among adult type 2 diabetic patients at public hospitals in Hadiya Zone, Southern Ethiopia. PLoS ONE. 2023;18(3):e0282962. 10.1371/journal.pone.0282962. 36952453 10.1371/journal.pone.0282962 PMC10035868

[22] Poonoosamy J, Lopes P, Huret P, Dardari R, Penfornis A, Thomas C, et al. Impact of Intensive Glycemic Treatment on Diabetes Complications-A systematic review. Pharmaceutics. 2023;15(7). 10.3390/pharmaceutics15071791. 10.3390/pharmaceutics15071791 PMC10383300 37513978

[23] Bin Rakhis SAS, AlDuwayhis NM, Aleid N, AlBarrak AN, Aloraini AA. Glycemic Control for type 2 diabetes Mellitus patients: a systematic review. Cureus. 2022. 10.7759/cureus.26180. 35891859 10.7759/cureus.26180 PMC9304683

[24] Care D, Suppl SS, Brown FM, Bruemmer D, Collins BS, Hilliard ME, et al. Glycemic Targets : Stand Care Diabetes. 2023;46(January):97–110. 10.2337/dc23-s006.

[25] Alteman RAAAH. The prevalence and determinants of poor glycemic control among adults with type 2 diabetes mellitus in Saudi Arabia. Diabetes, Metab Syndr Obes Targets Ther. 2018;15–21. 10.2147/dmso.s156214 10.2147/DMSO.S156214 PMC5797462 29430192

[26] Yosef T. Poor Glycemic Control and its contributing factors among type 2 diabetes patients at Adama Hospital Medical College in East Ethiopia. 2021;3273–80. 10.2147/dmso.s321756 10.2147/DMSO.S321756 PMC8289306 34290512

[27] Salama MS, Isunju JB, David SK, Muneza F, Ssemanda S, Tumwesigye NM. Prevalence and factors associated with alcohol consumption among persons with diabetes in Kampala, Uganda: a cross sectional study. BMC Public Health. 2021;21(1):1–10. 10.1186/s12889-021-10761-5. 33853561 10.1186/s12889-021-10761-5 PMC8045270

[28] Weir CB, Jan A. StatPearls. Treasure Island (FL): StatPearls Publishing; 2024. https://www.ncbi.nlm.nih.gov/books/NBK541070/. BMI Classification Percentile And Cut Off Points. [Updated 2023 Jun 26]. 31082114

[29] Dubale M, Gizaw K, Dessalegn D. Magnitude and predictors of poor glycemic control in patients with diabetes at Jimma Medical Center, Ethiopia. Sci Rep. 2023;13(1):1–11. 10.1038/s41598-023-42774-y 10.1038/s41598-023-42774-y PMC10518326 37743416

[30] Bitew ZW, Alemu A, Jember DA, Tadesse E, Getaneh FB, Seid A et al. Prevalence of Glycemic Control and factors Associated with Poor Glycemic Control : a systematic review and Meta-analysis. 2023. 10.1007/978-1-4615-9110-8_12 10.1177/00469580231155716 PMC10071101 36852627

[31] Muringo M, Mutai J, Gachoh i J. Factors Associated with glycated hemoglobin levels > 6.5% among Diabetic patients attending Kenyatta National Hospital, Kenya. J Diabetes Mellitus. 2021;11:10–25. 10.4236/jdm.2021.111002.

[32] Marjanović M, Đido V, Lang VB, Martinović Ž, Ovčina A. The Association of Clinical Characteristics and Lifestyle habits with poor glycemic control in patients with type 2. Diabetes Mellitus. 2021;3(1):79–84. 10.1016/S0140-6736(12)60283-9.

[33] Hamid A, Dawson AZ, Xu Y, Egede LE. Independent Correlates of Glycemic Control among adults with diabetes in South Africa. Int J Environ Res Public Health. 2024;21(4):486. 10.3390/ijerph21040486. 38673397 10.3390/ijerph21040486 PMC11050191

[34] Id SN, Birhan N, Amare F, Id GM. Rate of glycemic control and associated factors among type two diabetes mellitus patients in Ethiopia : a cross sectional study. 2021;177:1–12. 10.1371/journal.pone.0251506 10.1371/journal.pone.0251506 PMC8112661 33974654

[35] Brown FM, Bruemmer D, Collins BS, Hilliard ME, Isaacs D, Johnson EL et al. Pharmacologic approaches to Glycemic Treatment : standards of Care in Diabetes — 2023. 2023;46(January):140–57. 10.2337/dc23-s009 10.2337/dc23-S009 PMC9810476 36507650

[36] Awang H, Muda R, Rusli N. Epidemiology of poor glycemic control among patients with type 2 diabetes Mellitus in Terengganu State of Malaysia. 2022;4(5):89–94. 10.24018/ejmed.2022.4.5.1499

[37] Dauod AS. Glycemic control among type 2 diabetic patients attending the Family Medicine Health Center and the Diabetic Health Center in Erbil, Iraq : a comparative study. 2018;22(3). 10.24018/ejmed.2022.4.5.1499

[38] Fredrick C, Otieno T, Mikhail K, Acharya J, Muga N, Ngugi E, Njenga. Suboptimal glycemic control and prevalence of diabetes-related complications in Kenyan population with diabetes: cohort analysis of the seventh wave of the International Diabetes Management practices Study (IDMPS). Endocr Metabolic Sci. 2021;3:100093. 10.1016/j.endmts.2021.100093.

[39] Abadiga A, Mosisa M, Etafa G. W. Magnitude and prOedictors of poor glycemic control among patients with diabetes attending public hospitals of Western Ethiopia. PLoS One. 2021;16(2):e0247634. 10.1371/journal.pone.0247634 10.1371/journal.pone.0247634 PMC7906479 33630936

[40] Sendekie AK, Teshale AB, Tefera YG. Glycemic control in newly insulin-initiated patients with type 2 diabetes mellitus: a retrospective follow-up study at a university hospital in Ethiopia. PLoS ONE. 2022;17(5):e0268639. 10.1371/journal.pone.0268639. 35617250 10.1371/journal.pone.0268639 PMC9135271

[41] Choe SA, Kim JY, Ro YS, Cho SI. Women are less likely than men to achieve optimal glycemic control after 1 year of treatment: a multi-level analysis of a Korean primary care cohort. PLoS ONE. 2018;13(5):e0196719. https://doi.org/10.1371%2Fjournal.pone.0196719. 29718952 10.1371/journal.pone.0196719 PMC5931663

[42] Joung KI, Jung GW, Park HH, et al. Gender differences in adverse event reports associated with antidiabetic drugs. Sci Rep. 2020;10:17545. 10.1038/s41598-020-74000-4. 33067519 10.1038/s41598-020-74000-4 PMC7567832

